# Time perception and autistic spectrum condition: A systematic review

**DOI:** 10.1002/aur.2170

**Published:** 2019-07-23

**Authors:** Martin Casassus, Ellen Poliakoff, Emma Gowen, Daniel Poole, Luke Anthony Jones

**Affiliations:** ^1^ Division of Neuroscience and Experimental Psychology, School of Biological Science University of Manchester Manchester United Kingdom

**Keywords:** timing, time perception, autism, systematic review, temporal order judgements, temporal sensitivity, prospective timing, scalar expectancy theory

## Abstract

Problems with timing and time perception have been suggested as key characteristics of autism spectrum condition (ASC). Studies and personal accounts from clinicians, parents, caregivers, and self‐reports from autistic people themselves often refer to problems with time. Although a number of empirical studies have examined aspects relating to time in autistic individuals, there remains no clear consensus on whether or how timing mechanisms may be affected in autism. A key reason for this lack of clarity is the wide range of timing processes that exist and subsequently the wide range of methodologies, research paradigms, and samples that time‐based studies have used with autism populations. In order to summarize and organize the available literature on this issue, a systematic review was conducted. Five electronic databases were consulted. From an initial 597 records (after duplicates were removed), 45 papers were selected and reviewed. The studies are reviewed within different sections based on the different types of timing ability that have been explored in the neurotypical (NT) population: time sensitivity, interval timing, and higher‐order time perception. Within each section cognitive models, methodologies, possible clinical implications, and research results are discussed. The results show different consistency across studies between the three types of timing ability. The highest consistency of results showing atypical time perception abilities is found in high‐level time perception studies. It remains unclear if autism is characterized by a fundamental time perception impairment. Suggestions for future research are discussed. ***Autism Res** 2019, 12: 1440–1462*. © 2019 International Society for Autism Research, Wiley Periodicals, Inc.

**Lay Summary:**

This systematic review examines the different types of timing and time perception behavior that have been investigated in autism. Overall, there are a number of studies that show differences between autistic and non‐autistic individuals, but some studies do not find such differences. Group differences are more consistent across studies using complex tasks rather than simpler more fundamental timing tasks. We suggest that experiments across a range of timing tasks would be fruitful to address gaps in our knowledge.

## Background

Autism spectrum condition (ASC, termed autism in this article) is a neurodevelopmental condition characterized by deficits in social communication and interaction, as well as repetitive behavior and restricted interests [DSM‐V; American Psychiatric Association (APA), 2013]. Additionally, autism is often accompanied by unusual sensory experiences affecting individual or multiple modalities [Simmons et al., 2009], and altered motor behavior such as poor eye hand coordination and unstable balance [Gowen & Hamilton, 2013]. It has been suggested that disorders in timing and/or time perception may be a key characteristic, or cause of, some of the behavioral and cognitive impairments in autism [Allman, DeLeon, & Wearden, 2011; Allman & DeLeon, 2009]. However, it remains unclear the exact type of timing that is affected in autism and the impact that altered timing may have on the atypicalities that characterize the condition. Additionally, different timing abilities are anchored in different cognitive processes, but the evidence from these different (although likely related) abilities is often taken as a unique process suggesting a generalized impairment in time perception. This represents a source of imprecision that needs to be addressed, since characterizing a heterogeneous condition such as autism requires very precise terminology, well‐defined cognitive mechanisms, and strong methodologies in order to improve the reliability of findings. This review seeks to evaluate the current evidence of whether time perception impairments exist in autism, and if so, which types of timing and temporal processing are affected. This review will also discuss how such deficits could produce the behavioral or cognitive atypicalities seen in autism.

The terms and processes around time perception research (timing, time perception, temporal processing, etc.) are often used inconsistently, without consideration of the time scale under study, or the complexity in terms of the cognitive demands the tasks involve. Broadly speaking *timing* is the coordination of action or thought to respond to time critical events in the environment. This includes predicting when events will occur in time and the timing of behavior to occur at an optimal moment. Time *perception* refers to more specific cognitive skills (although distinguishable between them), such as the perception of the duration of an event or stimuli, the temporal order of stimuli, and having a sense of how quickly or slowly time seems to be passing. Other higher order abstractions cover a more general understanding of the passage of time, one's location in it, events occurring in certain temporal orders, and that objects change as a function of time. These higher order concepts about time may well be subserved by the more specific skills of time perception, although experimental evidence of this is scarce. Indeed, the relationships between the different types of timing are poorly understood even in neurotypicals (NTs). This review presents the different models used in time perception research in autism, provides specific and clearly distinguishable definitions of time perception for each model defining them and providing the corresponding evidence from those studies.

Self‐reports from autistic people, as well as reports from those who have regular contact with them (parents, teachers, and clinicians), often include difficulties with a sense of time. For instance, from Donna Williams an individual with Asperger syndrome who wrote a book of her inside view: “for me, a problem with sequencing is also about sense of time and the continuity (or lack of it) in my sense of personal history” [Williams 1996, as cited in Boucher, 2001, p. 165] or a report by a clinician Lorna Wing [as cited in Boucher, 2001, p. 88], who highlights (among other things) “The difficulties lie in comprehending the passage of time and linking it with ongoing activities…” Although these quotes illustrate that autistic individuals experience difficulties with timing and time perception, they cannot be interpreted as proof that autistic individuals have an atypical perception of duration. In fact, it remains unclear whether the difficulties such as those presented in the quotes, are “simply” a higher order understanding of time as an organizer of events, or whether these problems are caused by a fundamental perceptual problem of representing durations (or both). Here, we conducted a systematic review of the evidence for both higher order and fundamental timing deficits in autism.

**Figure 1:**
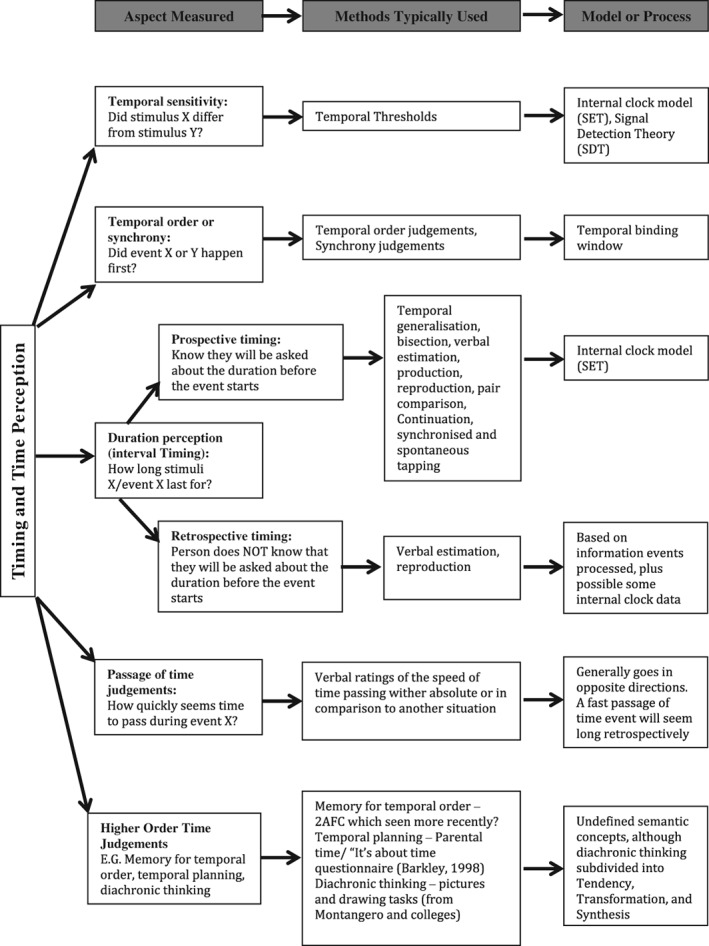
Common classification of timing abilities explored in neurotypical population.

The review is structured around the different types of timing ability that have been explored in the NT population (Fig. 1). Time perception studies have used a wide range of procedures involving different cognitive process, such as attention, working memory, executive function, an do forth (e.g., working memory load is highly relevant in interval timing, but not as strong in time sensitivity). Hence, this review is structured in three levels of complexity according to the increasing cognitive load demanded by the tasks used in the studies: time sensitivity, interval timing, and high‐level temporal processing. Within each section we (a) define the type of timing in question and the proposed cognitive mechanisms (where they exist), (b) describe the possible functional significance in relation to autism, (c) outline the results of studies that have examined autistic performance for that particularly timing ability, (d) present the time ranges used in the tasks, and (e) identify any gaps in empirical findings.

This review differs from existing reviews in the literature [Allman & Falter, 2015; Allman & Meck, 2012; Boucher, 2001; Chan, Langer, & Keiser, 2016; Stevenson et al., 2015] in that it is the first systematic review on time perception in autism. In addition, we have classified a wide range of timing behaviors in a hierarchical level of complexity according to the cognitive models they are based on and tasks used. Thus, in addition to autism researchers this review should be useful to anyone approaching the timing field for the first time, and/or has an interest in exploring a particular condition (not just autism) and its relationship with timing and time perception.

## Methods

A systematic search was carried out according with the guidance in the PRISMA statement [Moher et al., 2009]. The literature search was conducted during March 2017, looking for research papers published in peer‐reviewed journals without including any restriction in terms of year of publication. Web of Science, PubMed, Scopus, Wiley Online Library, and PsycINFO were consulted in the search using the term Autis* (so we picked up any derivation as autism, autistic, etc.) and its combination with *time perception* or *temporal order judgment*, or *time sensitivity*, or *temporal binding window* or interval tim* or prospective tim*. As exclusion criteria in the search the articles containing the terms schiz*or attentional deficit or hyperactivity were used. The reason for the exclusion criteria was that many articles about these conditions include autism in their conclusion and *autism* related terms in their abstracts or key words. Additionally, papers were included from suggestions obtained from research meetings.

Following the search, 596 papers were selected for abstract screening and another one was added at the end of the process [Jones et al., 2017 published online on 20th of April]. Three of the authors of this review screened the titles and abstracts independently and selected 76 for full reading, using the following inclusion criteria: (a) article was a research article (no reviews or theoretical works); (b) including at least one direct measurement in time perception in autism using any of the following methodologies: qualitative, psychophysics, questionnaires, neuropsychological tests, physiological measures, neuroimaging, or neurophysiology. In addition, the exclusion criteria were as follows: (a) research articles only about time or only about autism; (b) research articles about time perception and other conditions, disorders or pathologies including autism, but without presenting the autism data separated from the other conditions; and (c) articles about the use of the calendar in autism. The latter were excluded because they represent either a well‐mastered algorithm for date calculation or highly specified mathematical abilities, rather than time perception. Finally, one more study was included following a peer‐review suggestion.

**Figure 2:**
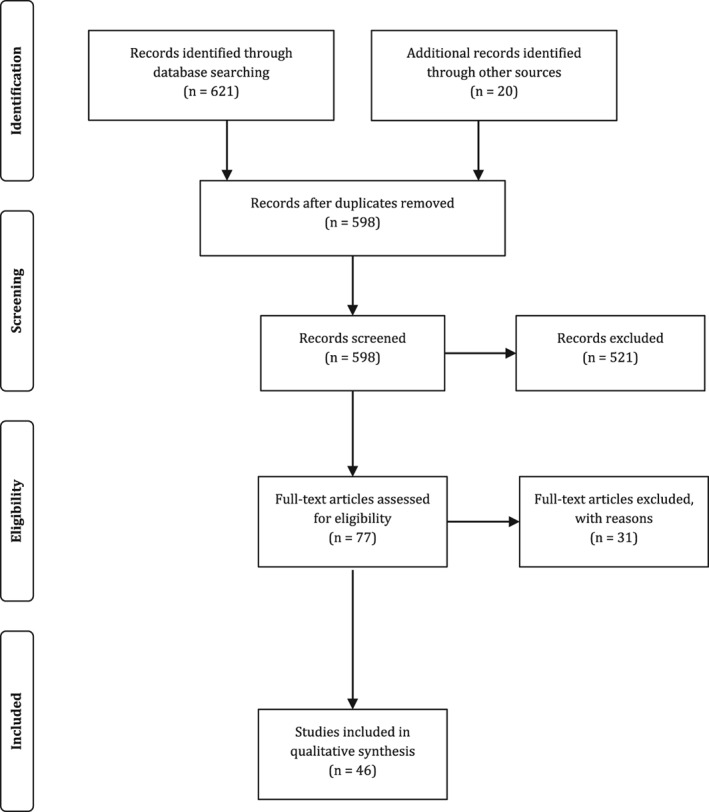
Flow diagram of the paper selection process (modified from Moher et al. [2009]).

From the 76 candidate papers, 31 were excluded. Twenty articles were excluded because they were not time perception research (but about other cognitive processes), or because the time perception data were not distinguishable from other cognitive processes. Five articles were excluded because they did not have an autism sample or the autism data were mixed with other conditions. A further two studies were excluded, because they covered circadian timing which is an area of research was not targeted in the current review (for readers interested in this area, interesting approaches can be found in Nicholas et al. [2007] and Hare, Jones, & Evershed [2006]). Finally, four articles were discarded because they were not research articles (Fig. 2).

The final 46 articles included in this review were separated by the type of timing ability investigated and are presented in the relevant section below. In addition, because the perception of durations changes during development [Droit‐Volet, Meck, & Penney, 2007] and autism follows a heterogeneous developmental trajectory [Fountain, Winter, & Bearman, 2012], we have separated the research into adults and children.

Finally, we use identity first language throughout this article in line with the preferences of autistic people identified in Kenny et al. [2016].

## Results

### *Time Sensitivity*

Temporal sensitivity measures a person's ability to respond to time or temporal stimuli in the environment. Unlike other perceptual processes, there is no information stream being transduced into an electrical–chemical signal by a sensory organ. Instead, the temporal characteristics of external stimuli have to be extracted by mechanisms within the organism. Nonetheless, we can still apply measurements of sensitivity that we would use in other sensory domains. Typically, time sensitivity research works in the millisecond range can be divided into temporal thresholds, temporal order/simultaneity judgements, and mismatch negativity (MMN) studies using electroencephalography (EEG).

Overall, 26 studies have researched time sensitivity, which we present according to the methodological approach.

#### *Temporal thresholds*

Evidence of a pure timing impairment in autism would come from studies using a fundamental test of temporal discrimination, for example, the measurement of thresholds for identifying when stimuli differ in temporal characteristics. A common method to research thresholds is a *staircase procedure*, where a standard time (of a fixed duration) is presented along with a comparison stimulus (variable durations). Then the participant identifies which of the two stimuli was longer. If the response is correct, the next trial increases in difficulty (less difference between stimuli) and if the response is wrong, then the next trial decreases in difficulty. A typical temporal threshold procedure lasts for 50 trials and the threshold is estimated by averaging the last 20 trials [see Jones, Poliakoff, & Wells, 2009 for a detailed example; and Treutwein, 1995 and Leek, 2001 for a further discussion of adaptive methods]. Time sensitivity research has shown differences in the ability to discriminate durations depending on the stimulus used in the task [see Rammsayer, 2010], which makes it difficult to compare the threshold values from one task with another. The most common stimuli used are filled durations (continuous tones), empty durations (delimited by a stimulus at the beginning and end; short beep–silence interval–short beep) or gap durations (a discontinuity; continuous tone–silence gap–continuous tone) (see Bhatara, Babikian, Laugeson, Tachdjian, and Sininger [2013], for an example of the latter).

Atypicalities with temporal thresholds may lead to a wide range of difficulties. If someone has very high thresholds (reduced temporal sensitivity), they would perceive two stimuli with different temporal characteristics as equal, while others would describe them as different. These differences may lead them to perceive the world as overwhelming, since different stimuli may overlap or disjoint.

Five studies have examined temporal thresholds in autism using different variants of the methodology described above (Table 1). Among the three studies conducted in children, two of them found no differences between autistic and NT samples and one showed reduced time sensitivity in autistic adults. Mostofsky et al. [2000] found no differences between autistic children and matched controls in auditory thresholds using empty intervals and an abbreviated threshold procedure. Jones et al. [2009] replicated the findings from Mostofky's study in a larger sample of children, but applying different methodology. Jones, Happé, et al. [2009] used a *More Virulent* PEST, with a more complex stimulus than Mostofsky (two cartoons of a dinosaur making a “funny sound,” as described by the authors) and provided feedback after each trial (correct or incorrect). In contrast, Bhatara et al. [2013] concluded that autistic children have impairments in auditory time sensitivity thresholds using a gap detection task, including feedback after each trial. The inconsistency in the findings between the three studies in autistic children could be due the different methodologies used.

**Table 1:** Temporal Sensitivity Measure by Thresholds

		Sample		Modality	Tasks	Findings	Commentaries
		ASC	NT				
Mostofsky, Goldberg, Landa, and Denckla [2000]	*n*	11	17	Auditory	Temporal thresholds: Empty intervals	No difference in thresholds between groups	Small sample Presence of outliers
	Age	13.3 (6.8–17.8)	12.5 (8.3–16.7)				Not a full threshold procedure
	IQ	101 (81–132)	105 (80–133)				
Jones, Poliakoff, and Wells [2009]	*n*	72	48	Auditory	Temporal thresholds: Filled intervals	No differences between groups in duration discrimination	A dinosaur is a more complex stimuli than the classic auditory paradigm
	Age	15.6 (5.7)	15.6 (5.9)				
	IQ	87.79 (17.32)	89.33 (21.53)				
Bhatara et al. [2013]	*n*	12	15	Auditory	Gap detection thresholds: Gap detection	Higher gap detection thresholds in ASC (15 msec) versus NT (5 msec)	Small sample
	Age	10.42 (1.92)	12.83 (1.75)				Lower verbal IQ in ASC (*P* < 0.01)
	VIQ	93 (16)	111 (13)				
	PIQ	99 (16)	105 (15)				
Kargas, López, Reddy, and Morris [2015]	*n*	21	21	Auditory	Temporal thresholds	Higher thresholds and higher variability in ASC	The authors warned that the SBRI scale of ADOS is not the best for measuring repetitive and restrictive behaviors
	Age	30.3 (10.4)	29.5 (11.4)				
	IQ	109.5 (18.3)	115.9 (10.6)				
						No correlation between SBRI^a^ scores and duration discrimination in ASC	
Poole, Gowen, Warren, & Poliakoff [2015], Poole, Couth, Gowen, Warren, & Poliakoff [2015]	*n*	18	18	Tactile	Temporal thresholds: Gap detection	No differences in tactile thresholds	Small sample
	Age	29.8 (8.1)	29.1 (7.2)				
	IQ	118.3 (9.9)	117.6 (13.4)				

a Stereotyped behaviors and restricted interests.

The two studies in adults also used different threshold procedures and sensory modalities. Kargas et al. [2015] compared auditory temporal thresholds between autistic adults and matched controls, finding higher thresholds in autism and no correlations between thresholds and Autism Diagnostic Observation Schedule (ADOS) scores on the Stereotyped Behaviors and Restricted interests scales. The other study in autistic adults [Poole, Gowen, et al., 2015] found no group differences examining tactile gap detection thresholds using a two‐interval procedure. Interestingly, a previous study using the same procedure [Poole, Couth, et al., 2015] found that higher tactile thresholds were associated with higher autistic traits in a NT sample.

Overall, temporal thresholds have been researched using different sample characteristics, methodologies, sensory modalities, and threshold calculations. In children, two studies show no differences and one shows differences between autistic and NT samples. In adults, the results are also mixed with one study showing differences and one showing comparable performance. Some studies have included the presentation of feedback, which is a factor that could affect performance (by providing a learning cue, and/or prompting an emotional response). Future research should attempt to replicate these findings, assessing each modality using full threshold procedures. In addition, studying filled and unfilled auditory thresholds in autistic adults would be useful to see if the findings in children remain in older ages. No studies have been conducted in tactile thresholds in children and no studies in either children or adults have been conducted on visual temporal thresholds. The latter is important because visual and auditory thresholds differ [Jones, Poliakoff, & Wells, 2009], so conclusions from one modality are not necessarily true for the other.

### *Temporal Order Judgments, Temporal Synchrony, and Temporal Binding Window*

**Figure 3:**
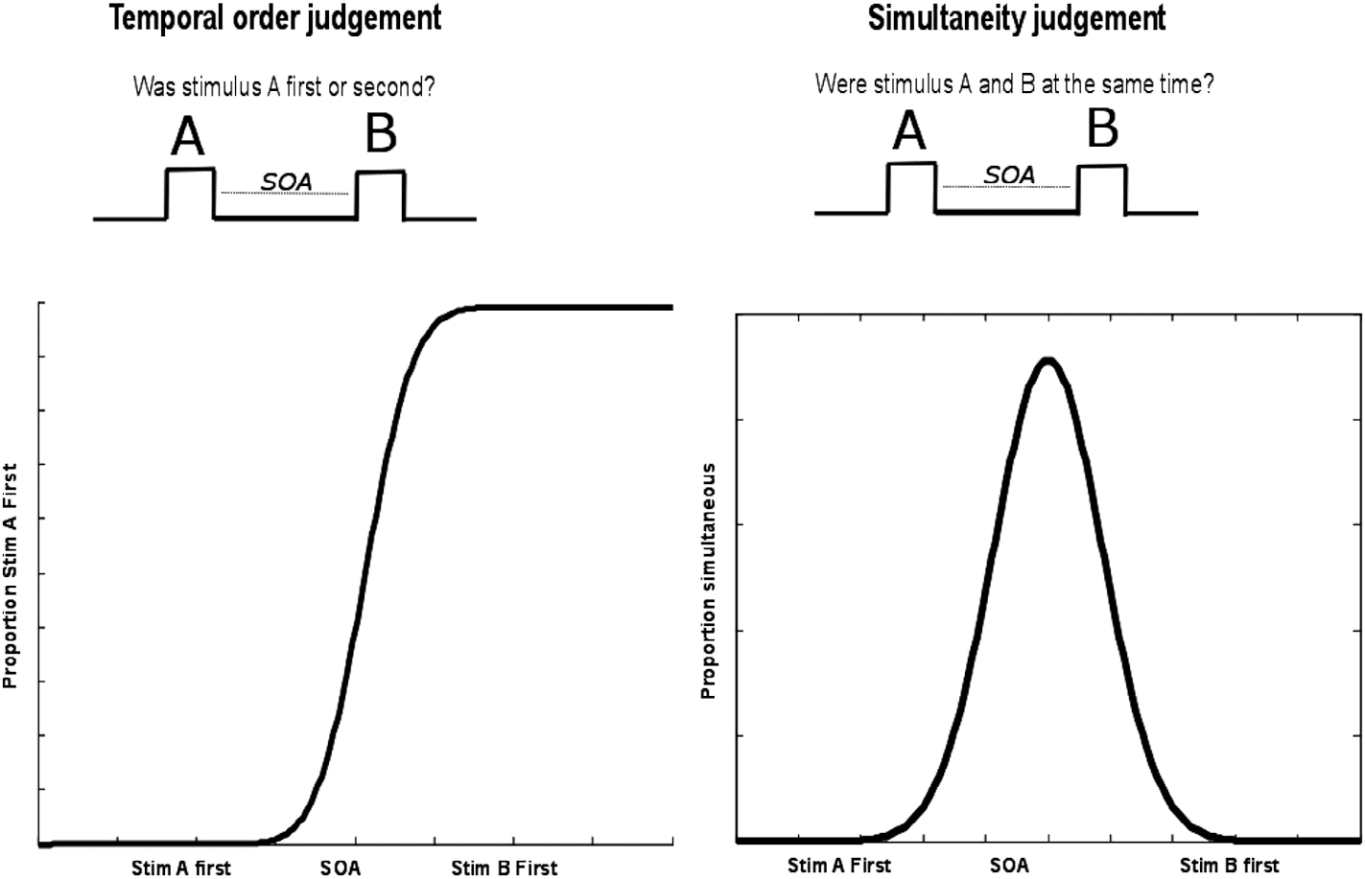
SJ and TOJ tasks involve detecting a temporal discrepancy, a TOJ involves additional processes such as identifying which of the two stimuli arrived first [Binder, 2015]. Two indices often used in the literature are the point of subjective simultaneity (PSS) and just noticeable difference (JND), which referred to the point where two stimuli are perceived as simultaneous and the smallest different between two stimuli to be judged as different respectively.

Another dimension of time sensitivity is discriminating whether events presented close in time are simultaneous, or preceded/succeeded by one another; that is simultaneity judgment (SJ) and temporal order judgment (TOJ) (Fig. 3). The measure of time sensitivity associated with this ability of separating stimuli in time is temporal acuity (TA) [Poole, Gowen, Warren, & Poliakoff, 2016]. Related to TA, is the concept of a temporal binding window (TBW) where information from the different sensory modalities (e.g., in multisensory tasks) are integrated only if they occur in temporal proximity to each other (see Wallace & Stevenson [2014] for a review in developmental disorders). A difficulty with TA may lead to problems with understanding which events in the world have been caused by our own action, termed a sense of agency [Gallagher, 2000; Jeannerod, 2003]. Differences in making cause–effect attributions could lead to superstitious thinking or self‐referred logical thinking, commonly described in autism. Additionally, it may have implications in providing continuity to one's own experience and may be related to difficulties with prospective timing (see below).

Fifteen studies have examined TOJ and SJ in different modalities, using a variety of methodologies. In 12 studies with autistic children, seven used TOJ tasks and five used other tasks. Regarding the TOJ studies, three reported reduced TA in autistic children, one showed no differences between autistic children and matched controls and three studies showed mixed results (reduced temporal sensitivity in autistic children in some tasks/measures, but no differences in others). Wada et al. [2014] demonstrated reduced temporal sensitivity in the tactile modality, whereas, Puts, Wodka, Tommerdahl, Mostofsky, and Edden [2014] found no differences between autistic children and matched controls (Table 3). Comparable performance between autistic and control groups in children has also been reported in the visual modality by Kwakye, Foss‐Feig, Cascio, Stone, and Wallace [2011], although the same study found reduced TA in the auditory modality in autistic children. Most bimodal research has focused on audiovisual interactions. De Boer‐Schellekens et al. [De Boer‐Schellekens, Eussen, & Vroomen, 2013; De Boer‐Schellekens, Keetels, Eussen, & Vroomen, 2013] reported reduced temporal sensitivity in autistic children using a TOJ task in visual and audiovisual modalities, respectively. Audiovisual interactions were also examined by Stevenson et al. [2014] and Noel, De Niear, Stevenson, Alais, and Wallace [2016] who reported no differences between autistic children and matched controls for simple stimuli (flash‐beep) and complex stimuli (tool), but a wider TBW in autistic children for speech stimuli. In contrast, Foss‐Feig et al. [2010] found a wider TBW in autism working with a different task (Flash‐Beep illusion; see Shams, Kamitani, & Shimojo [2000] for a full explanation of the illusion).

Temporal integration of audiovisual stimuli has a relevant role in communication (e.g., in speech) and has been explored using tasks other than TOJ or SJ. Bebko, Weis, Demark, and Gomez [2006] used a preferential looking paradigm, concluding that autistic children have an impaired ability to process asynchronous linguistic stimuli. Irwin, Tornatore, Brancazio, and Whalen [2011] showed that autistic children rely more on auditory than visual information in audiovisual speech stimuli, and Grossman, Steinhart, Mitchell, and McIlvane [2015] showed that autistic children dedicate less time looking at the mouth region of the face in speech stimuli. Using a different bimodal approach (visual‐tactile), Greenfield, Ropar, Smith, Carey, and Newport [2015] worked with the “rubber hand illusion” showing no differences in sensitivity to asynchrony between autistic and control participants matched for mental age, but differences when compared to chronologically matched controls (Table 2).

**Table 2:** TOJ, SJ, and TBW in ASC Children and Adolescents

		Sample		Sensorial Modality	Tasks	Findings	Commentaries
		ASC	NT				
De Boer‐Schellekens, Eussen, & Vroomen [2013], De Boer‐Schellekens, Keetels, et al. [2013]	*n*	16	16	Bimodal (A‐V) handclap speech flash/beep	TOJ^a^	Lower time sensitivity in ASC group	Small sample
	Age	19.3 (2.4)	19.3 (1.3)				
	IQ	106.2 (14.1)	106.6 (8.4)			No differences between conditions	
De Boer‐Schellekens, Eussen, and Vroomen [2013]	*n*	35	40	Visual	TOJ	Lower time sensitivity in ASC group	
	Age	18.8 (2.1)	18.8 (1.3)				
	IQ	103.2 (14.6)	107.9 (9.1)				
Kwakye et al. [2011]	*n*	35	27	Visual	TOJ	No differences in visual thresholds	
	Age	12.21 (2.7)	11.73 (2.4)	Auditory			
	IQ	102.9 (18.7)	109.5 (10.8)	Bimodal		Less temporal sensitivity in auditory stimuli in ASC	
					
						Larger TBW in ASC	
Puts et al. [2014]	*n*	27	54	Tactile	TOJ	No differences on TOJ tasks between groups	Differences between groups in IQ
	Age	10.7 (1.015)	10.08 (1.28)				
	IQ	103.14 (14.93)	117.33 (12.24)				
Stevenson et al. [2014]	*n*	32	32	Bimodal flash‐beep, tool, syllable	TOJ	Wider TBW in ASC for speech stimuli No differences for flash‐beep and tool stimuli	Differences between groups in verbal IQ
	Age	11.8 (3.2)	12.3 (2.3)		SJ^b^		
	IQ	57.5 (8.4)	53.7 (8)				
				
Wada et al. [2014]	*n*	10	10	Tactile	TOJ	Reduced temporal sensitivity in ASC	Small sample
	Age	11.8 (0.7)	11.7 (0.7)				
	IQ	100.7 (6.5)	101.6 (2.4)			No detriment in performance when hands‐crossed in ASC	
						
						
Noel et al. [2016]	*n*	26	26	Bimodal flash‐beep, tool, syllable	TOJ		
	Age	12.3 (3.05)	11.6 (3.79)		SJ		
	IQ	111.52 (14.73)	112.18 (7.56)			Wider TBW in ASC for speech stimuli. No differences in flash‐beep and tool	
					
Foss‐Feig et al. [2010]	*n*	21	17	Bimodal flash‐beep	Flash‐beep illusion	Wider TBW in ASC	
	Age	12.8 (2.61)	12.9 (2.2)				
	IQ	108.45 (18.7)	107.19 (9.3)				
Irwin et al. [2011]	*n*	13	13	Bimodal (A‐V)	Asynchrony	ASC sample performed similarly with mental age matched, but not with chronological age No differences between groups	Small sample
	Age	9.08	9.16				
			
					
Bebko et al. [2006]	*n*	16	15 DD/16 NH	Bimodal (A‐V)	Preferential looking	ASC only showed preferential looking for asynchronous non‐linguistic events	Small sample
	Age	5.49 (0.51)	4.88 (0.72) DD/2.36 (0.68) NH				
Greenfield et al. [2015]	*n*	29	29CA/29MA	Bimodal (A‐V)	Rubber hand illusion	ASC sample performed similarly with mental age matched, but not with chronological age	
	Age	12.64 (1.9)	12.18 (1.78) CA/7.88 (1.39)				
Grossman et al. [2015]	*n*	30	30	Bimodal (A‐V)	Eye‐tracking	Less gaze to in‐synch condition in ASC Less gaze time to mouth area in ASC	
	Age	11:10 (1:4)	12:5 (0.11)				
	IQ	104 (15.9)	109 (11.2)				

a Temporal order judgment.

b Simultaneity judgment.

TOJ studies in autistic adults have also produced mixed results. In Tommerdahl et al. [2008], the autistic sample exhibited higher tactile thresholds when the stimulus was applied to one hand, but comparable performance when the stimulus was applied to both hands. In contrast, Falter, Elliott, and Bailey [2012] found superior visual TA in autism and a negative correlation between autistic symptoms and thresholds. A different approach by Poole et al. [2016] examined bimodal pairings (auditory–visual, auditory–tactile, and visual–tactile) in a TOJ task, finding no differences in any of the conditions (Table 3), however, performance was correlated with sensory symptoms across the groups.

**Table 3:** TOJ and SJ in High‐Functioning ASC Adults

		Sample		Modality	Tasks	Findings	Commentaries
		ASC	NT				
Tommerdahl, Tannan, Holden, and Baranek [2008]	*n*	10	20	Tactile	Unilateral SJ and TOJ	Worse temporal sensitivity in ASC	Small sample
	Age	26.1 (6.3)	24.2 (6.1)				
	IQ	102.8 (17.7)	115.6 (7.1)	Tactile	Bilateral TOJ	Comparable temporal sensitivity	No correlation with symptomatology done
					
Falter, Elliott, and Bailey [2012]	*n*	16	16	Visual	SJ	Better temporal sensitivity in ASC	Small sample
	Age	24.2 (7)	26.2 (7.4)				
	IQ	114 (13)	112 (9)			Negative correlation between temporal thresholds and autistic symptoms	
						
						
						
Poole et al. [2016]	*n*	18	18	Bimodal dyads (A‐V, V‐T, A‐T)^a^	TOJ	No differences between groups in JND nor PSS for all the dyads	Small sample Differences reported in other studies in SOAs between 150 and 300 msec were under‐represented in the design
	Age	31 (8.43)	31.05 (8.71)				
	IQ	116.56 (9.7)	112.76 (7.56)				

a A‐V: auditory–visual; A‐T: auditory–tactile; V‐T: visual–tactile.

There is a trend for TA and multisensory temporal integration for socially relevant stimuli to differ in autistic children and adults, but care must be taken when interpreting these differences as timing deficits as they may be confounded by reduced attention to social stimuli in autism [Dawson et al., 2004]. A further issue is that many of the methodologies described in this section could be influenced by differences in response bias. For instance, on a SJ task autistic participants may be less conservative in the use of the “simultaneous” response in conditions of relative uncertainty. This situation would lead to an increased frequency of simultaneous responses across a range of SOA (Stimulus Onset Asynchrony) and the apparent conclusion that temporal sensitivity was reduced in autism (or that the TBW is widened; see Yarrow, Jahn, Durant, & Arnold [2011] for a more detailed discussion of this issue).

### *EEG and Time Sensitivity*

Four studies have attempted to measure time sensitivity using EEG. All these studies have worked with event‐related potentials (ERPs), specifically with MMN, where bigger wave amplitudes are interpreted as better time sensitivity, in terms of higher discrimination (for a deeper understanding of ERP in time processing, see Macar & Vidal [2004] and Ng & Penney [2014]). In children, two studies [Lepisto et al., 2005; Lepistö et al., 2006] have shown diminished MMN amplitude, so implying reduced temporal sensitivity. In contrast, the studies in autistic adults [Kujala et al., 2007; Lepistö, Nieminen‐von Wendt, Von Wendt, Näätänen, & Kujala, 2007] showed enhanced discrimination abilities in autistic adults compared with matched controls, in frontal and central‐line electrodes (Table 4).

**Table 4:** Mismatch Negativity Studies in Temporal Sensitivity in ASC

		Sample		Sensory Modality	Tasks	Findings	Commentaries
		ASC	NT				
Kujala et al. [2007]	*n*	8	10	Auditory	EEG: MMN	Enhanced time sensitivity in ASC	Small sample
	Age	27	30				
	IQ	106	112				
Lepistö et al. [2007]	*n*	9	10	Auditory	EEG: MMN	Enhanced time sensitivity in ASC	Small sample
	Age	27	30				
	IQ	VIQ: 104; PIQ: 108	VIQ: 113; PIQ: 116				
Lepistö et al. [2006]	*n*	10	10	Auditory	EEG: MMN	Diminished time sensitivity in ASC	Small sample
	Age	8.11	8.1				
	IQ	VIQ: 108; PIQ: 112	VIQ: 107; PIQ: 114				
Lepisto et al. [2005]	*n*	15	15	Auditory	EEG: MMN	Diminished time sensitivity in ASC	Small sample IQ differences between groups
	Age	9.4	9.4				
	IQ	PIQ: 95	115				
						

### *Summary of Time Sensitivity Studies*

Overall, several studies have researched time sensitivity in autism using different methodologies. In adults three studies showed enhanced time sensitivity, two studies show no differences between autistic and NT samples and two show reduced time sensitivity. Therefore, it is difficult to describe a clear trend for autistic adults in terms of their time sensitivity abilities. In children, 11 studies show reduced time sensitivity abilities in ASC, three show mixed results, and three show no differences between groups. Taking into account that (a) there is more consistency across studies in children/adolescents than adults; (b) there are no enhanced abilities reported in children, but they are present in adults; and (c) there are more studies in children reporting impaired abilities, it can be hypothesized that there may be a differential developmental trajectory between autistic and NT individuals (although it should be noted that there are more than twice as many studies available for children compared to adults). Developmental studies of time sensitivity abilities are needed to explore this hypothesis.

## Duration/Interval Timing

Interval timing is the perception of the duration of a stimulus or an event, allowing us to perceive how long a stimulus lasts for. It is crucial for our everyday interaction with the environment in predicting the timing of events. If impaired, one might become frustrated during waiting periods, as they are not predictable, and delayed in reacting to events. Additionally, impairment in this area may impact on conversation turn‐taking, and on social coordination, which requires a shared understanding of *when* an event will take place, therefore, atypicalities in interval timing may be related to predictability issues in autism. It has been suggested that these issues with interval timing may lead to repetitive behaviors as a possible strategy to parse time [Boucher, 2001; Allman, 2011]. In addition, one may have more difficulty anticipating the occurrence of daily events, or knowing how long one has been engaged in a particular activity, perhaps leading to the reported over‐reliance on time keeping devices, or strict schedules of wake, sleep, eating, and so forth that are reported in autism. Interval timing is also related to the ability to attribute cause and effect and our sense of agency (see Temporal Order Judgments, Temporal Synchrony, and Temporal Binding Window Section).

### *Prospective Timing*

**Figure 4:**
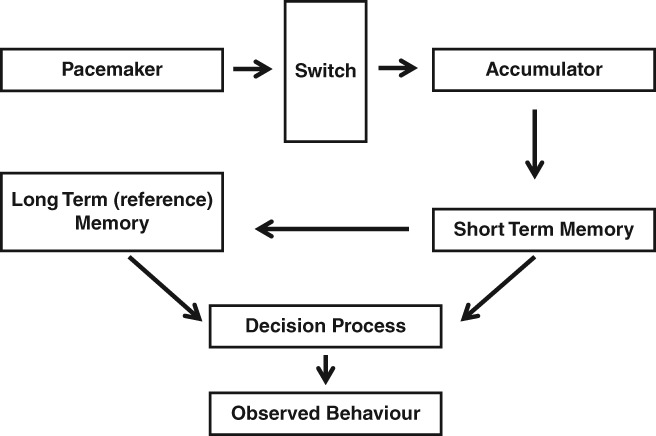
SET Model: When a duration is to be timed prospectively, the switch closes allowing pulses to flow from the pacemaker to the accumulator. At the end of the stimulus, the switch opens and pulses stop flowing. The amount of pulses or ticks accumulated is the subjective estimation of the stimulus duration. If this duration is important, or is to be used for future judgments it can then be stored in the reference or long‐term memory. The comparator can make similarity judgments between the current duration (contents of the accumulator) and previously experience duration (contents of the reference memory).

Prospective timing involves the judgment of stimulus duration when the participant is aware that such a judgment will be required. For example, the participant listens to a tone, and makes some judgment about its duration, or compares its duration with a reference duration. As the participant is aware that this judgment will be required, they will be ready to start timing the stimulus as it commences. Prospective timing judgements are commonly thought to be underpinned by an internal clock system, based on scalar expectancy theory [SET; Gibbon, 1977; Gibbon, Church, & Meck, 1984]. The system consists of three main stages, a clock stage (made up of a pacemaker, switch, and accumulator), a memory stage (consisting of short term and reference long‐term memory), and lastly a comparison or decision stage where different durations can be compared to each other (Fig. 4). Research in prospective time involves durations from the millisecond range to several seconds.

There is a large body of evidence supporting pacemaker‐accumulator clocks over other models [Wearden & Jones, 2007; Wearden, 2005; Wearden & Doherty, 1995]. For example, human judgments of duration increase linearly with an increase in stimulus duration (implying a monotonic accumulator process), timing sensitivity is very accurate with difference thresholds as little as 10 msec (dependent on the duration timed, as Weber's law, which states that the just noticeable difference remains a constant fraction of the mean, holds for duration judgments) and humans can make ordinality judgments about different durations. An additional quality of the SET model is that its operation is mathematically defined, such that one can use computer modeling of timing data in order to identify which component can explain differences in performance (an example of such modeling for temporal bisection is shown in Box 1, other tasks such as generalization and magnitude estimation can also be modeled with SET).

Box 1 **Computer modeling of temporal bisection**

The computer model of Temporal Bisection using the mathematics of SET [from Droit‐Volet et al., 2001].

The model calculates two differences:

1. D(s*,*t)*
  This is the absolute difference between the current trial duration, *t* (which is assumed to be timed without error, or negligible relative variability) and s* which is a sample drawn on each trial from the (Gaussian) memory distribution of the short anchor.
2. D(l*,*t)*
  The absolute difference between *t* and l* which is a sample drawn from the memory distribution of the long anchor.

If D(s*,t) ‐ D(l*,*t) < b* (where *b* is the threshold value), then the model responds “long.” If the difference is greater than *b* then the model responds “short” if D(s*,t) < D(l*,*t)* and responds “long” if D(s*,t) > D(l*,*t)*. Essentially, if the model cannot tell whether *t* is closer to the long or short anchor it responds “long.” Variability in the system is controlled by three main variables, “*c*” which controls the coefficient of variation of the memories of the long and short anchors, K* which controls the mean of the memory distributions (i.e., if the value of K* > 1 then the anchors are remembered systematically as being longer than they actually were, if K* < 1 then shorter), and “*b*” which controls the threshold.

#### *Comparison methods*

**Figure 5:**
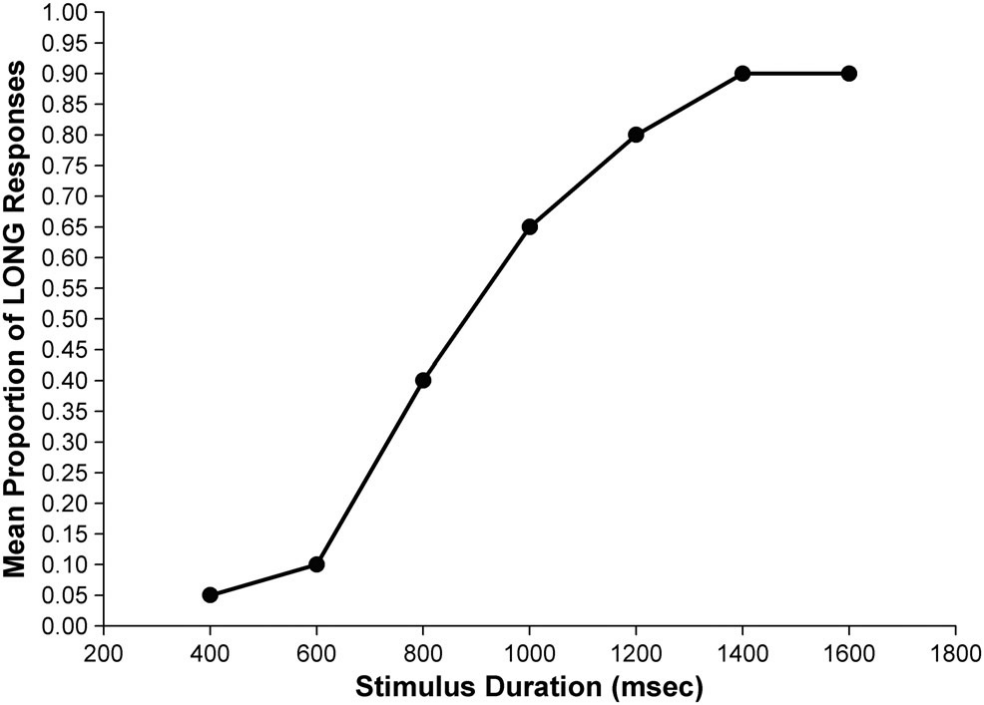
Psychophysical function in bisection tasks: Proportion of “Long” responses plotted against stimulus duration produces a typically sigmoidal psychophysical function, with “more similar to long” responses being at around zero at the shortest anchor and near 1 at the long anchor duration. The location of the bisection point (BP: 50% “long” responses) can characterize certain response biases, or memory effects either caused by individual differences or experimental manipulation. The BP is usually located at the arithmetic mean of the two anchors standards (in humans; Wearden, 1995). The difference limen (DL: temporal variability) can be calculated by taking half the differences between 25% and 75% “long” responses. The Weber ratio (WR, measure of temporal sensitivity) can be calculated by dividing the DL by the BP.

These tasks involve comparing a given stimulus with a previously given reference. The most common tasks are temporal bisection and temporal generalization. In temporal bisection, participants first learn to discriminate between two anchor durations, a long and a short duration. They are then given a range of durations that span (and include) these two anchors and are asked whether each was more similar to the long or short anchor. Common indices are bisection point (BP), difference limen (DL), and Weber ratio (WR). BP is the point in which a participant answers either long or short responses in same proportion. DL is the minimum difference between stimuli to be discriminated. WR measure of time sensitivity is based on the steepness of the curve (Fig. 5). A key prediction of SET is that the WR should remain constant when different durations are timed, called the scalar property, which can also be tested by looking for superimposition by plotting the psychophysical functions from two different anchor durations on the same relative scale as they should superimpose.

**Figure 6:**
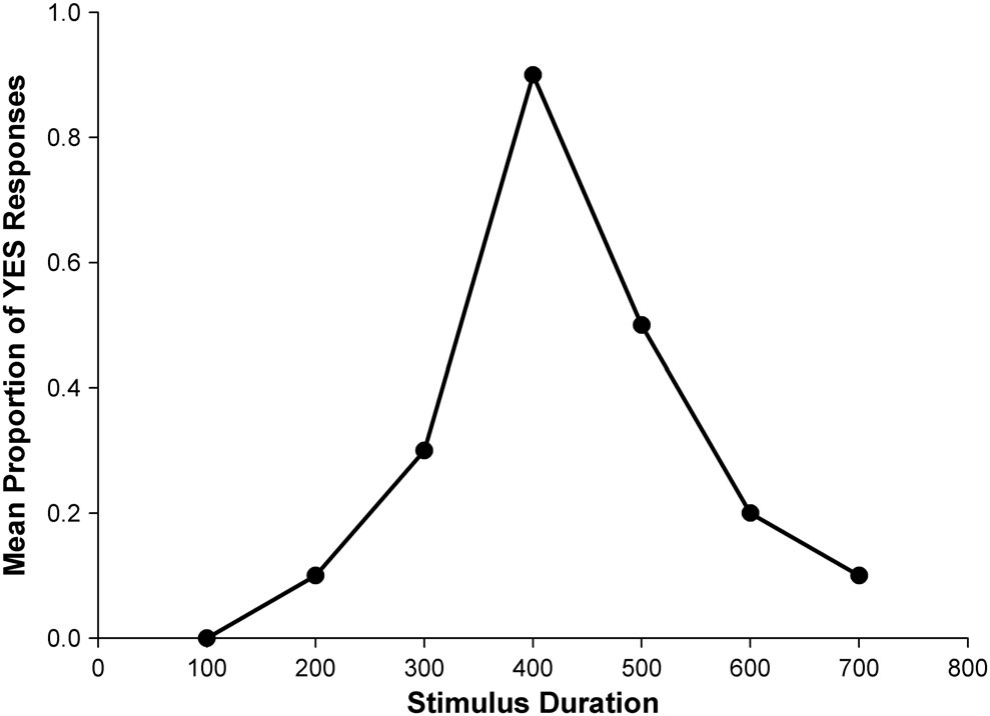
Typical temporal generalization function plotted where the maximum number of “yes” responses typically occurs when the comparison is identical to the standard duration, and decreases as the difference between them increases. The steepness of this function gives a measure of temporal sensitivity, and because comparisons both longer and shorter than the Standard are presented, the function allows for the identification of distortion or asymmetry in responding. These functions are typically slightly asymmetrical in normal adults, with more YES responses to comparisons longer than the standard than for comparisons that are shorter; i.e., adult have a slight tendency to confuse durations that are longer than standard with the standard more than durations that are shorter (see Wearden [1992] for full discussion).

In temporal generalization a “Standard” duration (e.g., a 800 msec tone) is presented several times, then a number of comparison durations are presented (400, 500,…, 900 msec, etc. in a random order), and after each comparison presentation the participant is asked “was that the Standard duration?” (yes/no; see Fig. 6). Data from generalization tasks can be tested for the scalar properties of interval timing.

Five studies have worked with prospective comparison methods in autism, with three in children and two studies in adults. All the studies with autistic children used a temporal bisection task and one also used a temporal generalization task. Allman et al. [2011] used two versions of the temporal bisection procedure to examine autistic children (durations of 1 and 4 sec, and 2 and 8 sec). In both versions of the task, the autistic group's BP was located at a shorter duration than the controls. Although in the 1–4 sec range this effect appears to have been driven by abnormal performance of the controls, because they were higher than expected, and the autistic participants' BP was in the range reported by previous experiments with NTs. There were no group differences in WR for the 1 and 4 sec anchors task. In the second version, the autistic group had significantly higher WRs than the controls, indicating reduced sensitivity. Both NT and autistic groups demonstrated superimposition, although this was less strong for the autistic group. In the autism group, shorter BPs were associated with worse scores for language and communication (measured with ADOS) and working memory.

Allman et al. [2011] used computer modeling on the bisection data. The model is from Wearden [1991], and previously used to model data from typically developing children [Droit‐Volet, Clément, & Wearden, 2001; Box 1]. This model fitted the data from the autism group and the controls well, for both anchor durations, with autistic individuals requiring a higher value of *c* (*which determines the memory variability*) to fit the data compared to the controls. In fact, the values obtained for the autism group were similar to those used to fit data from 5 year‐olds NTs, whereas the values for the control group were similar to that used to fit data from 8‐year olds (which are identical to adults). For the 1 and 4 sec anchor experiment the values of *K* (which determines the mean accuracy of the memory distribution of the anchors, see Box 1; for discussion of K* and reference memory function see Jones & Wearden [2003, 2004]) for both groups were similar to that of typically developing children in other studies. However, in the 2 and 8 sec anchor experiment the autistic group had a lower value of K*, suggesting that they systematically remembered the anchors as shorter than they actually were. This is similar to that seen with 3 year‐olds NTs [although on a generalization task: Droit‐Volet et al., 2001]. Although this is suggestive of developmental differences in autism, according to the authors themselves, one would need a much wider range of temporal tasks in order to characterize the amount and source of variance in the timing system.

Gil, Chambres, Hyvert, Fanget, and Droit‐Volet [2012] used a temporal bisection task with four different duration ranges, two long and two short, ranging from 0.5 to 16.63 sec. They found no differences between groups in any of measure (BP, DL, and WR). Consequently, Gil et al. [2012] concluded that autistic children have “the working raw material” for time perception and their day‐to‐day issues are probably due the integration of other cognitive processes (attention, memory, etc.) with temporal information to produce time judgments. However, as acknowledged by the authors, the results could have been influenced by a modification of the task to increase participants' sustained attention. A difference between Allman et al. [2011] and Gil et al. [2012] was that the modality of responses was different (key‐press and verbal response, respectively). Brodeur, Gordon Green, Flores, and Burack [2014] reported reduced performance of 15 low‐IQ autistic children compared to controls matched for mental age in temporal generalization and bisection tasks. In addition, multisensory cartoons (image plus sound) were used to present the task, so the results may reflect issues with multisensory perception rather than time perception.

Researching autistic adults, Falter, Noreika, Wearden, and Bailey [2012] used a temporal generalization task with visual, auditory, and audiovisual stimuli. Autistic individuals showed a clearer adherence to the scalar property than the control group, as well as the same effect of perceiving auditory durations as subjectively longer than visual ones as the controls [a well‐characterized phenomenon in NTs, e.g., Goldstone & Lhamon, 1972, 1974; Jones, Poliakoff, & Wells, 2009; Wearden, Todd, & Jones, 2006]. Signal detection analysis showed that the autistic group had reduced temporal discrimination compared to the controls, particularly for auditory stimuli. Lastly, the response criteria of the autism group was related to symptom strength in communication, the stronger the symptom strength the more conservative the response bias, that is, the less likely they are to identify the comparison as the Standard [Falter, Noreika, et al., 2012]. In contrast, Jones et al. [2017] found no differences between autistic adults and matched controls using a temporal bisection task and a set of stimuli of emotionally charged faces and wildlife images (Table 5). Although, the more complex nature of the stimuli used by Jones et al. [2017] have the benefit of being more ecological than simple beeps or flashes, they could increase the involvement of other cognitive processes, since it has been suggested that emotions affect our perception of duration, physiological arousal, attention, and working memory (for a discussion see Lake, LaBar, & Meck [2016]; Droit‐Volet and Meck, 2007).

**Table 5:** Prospective Comparison Tasks in ASC

		Sample ASC	NT	Task	Main conclusions	Commentaries
Allman et al. [2011]	*n*	13	12	Temporal bisection	Bisection point in ASC shorter than NT in two anchors (1–4 and 2–8 sec) No differences in WR in anchor 1–4 sec Higher WR in ASC in anchor 2–8 sec	Small sample Weak characterization of the control group
	Age	10.3	10.3			
	IQ	92.31	109.8			
Gil et al. [2012]	*n*	12	12	Temporal bisection	No differences in BP, DL or WR Good adjustment to scalar timing properties in both groups	Small sample Changes in the research paradigms were introduced to maintain participants' attention; however, this effect was not tested
	Age	13	13.21			
	IQ	94.37	101.45			
Brodeur et al. [2014]	*n*	15	15	Temporal Generalization	No group main effect, but group by duration main effect was reached	Small sample No computer modeling or signal detection theory applied in either task
	Age	10.74 (3.93)	6.46 (0.93)			
		CA 7.3 MA	CA 6.46 MA			
	*n*	15	15	Temporal bisection	Higher DL and BP in ASC No group main effect, but group by duration main effect was reached	Small sample No statistical comparisons of DL, BP, or WR
	Age	10.16 (3.93)	6.61 (0.78)			
	IQ	CA 6.19 MA	6.22 MA			
				
Falter, Elliott, and Bailey [2012]	*n*	18	19	Temporal generalization	Less temporal sensitivity in ASC Higher consistency in the responses between different time intervals	Small sample
	Age	25.3	26.1			
	IQ	112	113			
Jones et al. [2017]	*n*	20	26	Temporal bisection	No differences between groups in WR or BP	No computer modeling performed or signal detection theory
	Age	45.4	44			
	IQ	114.6	108.1			

#### *Estimation methods*

Estimation methods involve estimating a given duration and expressing it with some predefined behavior (writing, verbalizing, pressing, etc.). The most common tasks are verbal estimation (an answer in time physical units such as seconds or milliseconds), temporal reproduction (the participant recreates a given duration), and temporal production (the participant produces a duration from a given temporal target usually in second or milliseconds). Common indices in these tasks are accuracy (e.g., mean of the interval reproduce/verbally estimated/produce, divided by the reference duration) and consistency (variability measure, e.g., coefficient of variation). These measures allow comparison of performance between different references, overestimation, and underestimation of durations.

Eight studies have researched prospective time by estimation methods in autism, with five of them studying autistic children. Szelag, Kowalska, Galkowski, and Poppel [2004] compared seven autistic and NT children across a range of durations from 1 to 5.5 sec in visual and auditory modalities. Performance was worse in the autistic group who reproduced all the durations at around 3–3.5 sec, so they did not adjust to scalar timing. Importantly, the groups were not well matched on IQ, so differences could arise from IQ differences rather than autism diagnosis.

Wallace and Happe [2008] conducted a study using a stopwatch in tasks of verbal estimation, production, and reproduction in 25 autistic adolescents and matched controls, using durations from 2 to 45 sec. No differences were found between groups in the three tasks, but there was a trend for better performance in the time reproduction task in the autistic group. However, the authors acknowledge that the recruitment of savants could have been a factor affecting their results. In contrast, Maister and Plaisted‐Grant [2011] performed two time reproduction experiments in which participants pressed a key (instead of the researcher using a stopwatch as in Wallace & Happe [2008]). In their first experiment, they found impairments in short durations under 2 sec, and for the longest duration of the task (45 sec). In the second experiment, they only found differences in the extreme durations they used (0.5 and 45 sec). They also investigated the relationships between time reproduction and memory abilities. Short‐term memory was correlated with the error scores for short durations between 1 and 10 sec in both groups, but no statistical significance was found with the shortest duration of 0.5 sec. For long durations (>30 sec), a significant correlation was found between long‐term memory and time reproduction only in the NT group.

Brenner et al. [2015] compared the performance in a time reproduction task between autistic and matched control children and adolescents. Using times ranging from 4 to 20 sec, the authors observed poorer performance in the autistic group in accuracy and consistency, with the first index being associated with age, and the second with working memory. Recently, Karaminis et al. [2016] found that autistic children performed significantly worse than the matched control group in time reproduction in terms of accuracy, but not consistency. Additionally, they worked with a discrimination task showing higher thresholds in the autistic group (similar to a younger group 6–7 years old). The authors hypothesize that this could be explained by reduced integration of a central tendency prior (bias to the mean duration of previous stimuli), more than due a developmental delay (see Pellicano and Burr, 2012, and commentaries for further discussion). To assess the latter, the authors employed Bayesian modeling, finding less influence of prior knowledge in autism in comparison with NT. Finally, all groups of children showed underproduction of the duration in the time reproduction task, a phenomenon that did not appear in the adult group, but that has been described for children in previous studies [McCormack, Brown, Maylor, Darby, & Green, 1999].

In adults, findings using estimation methods are mixed. Gowen and Miall [2005] used a blend of reproduction and classical synchronized and continuation tapping, finding that the autism group had greater absolute error and greater stimulus asynchrony on the synchronization task, but without differences in the coefficient of variation. Hypothetical differences in the clock speed would show only a difference (if any at all) between the two groups on the continuation task (continuing to tap without a beat) and not on the synchronization task (tapping in time to a beat). If two groups differed in internal clock speed by a factor of two, then they could both still show identical synchronization, with one group simply timing their tapping after *n* ticks and the other after *2n* ticks of the internal clock. In the continuation task, one might expect to see some difference as they are no longer being presented with an external time marker (the beep) to which to calibrate their responses. It is possible that these findings indicate greater impairments in motor rather than clock variance, but this would need a full Wing and Kristofferson [1973] type design to tease apart these alternatives (see Wearden and Jones, 2013 for a detailed explanation of this issue). To date, no study has separated perceptual clock timing from motor timing in autism. Given the frequent occurrence of movement difficulties in autism [Gowen & Hamilton, 2013], this is an important issue to investigate.

Using a time reproduction task, Martin, Poirier, and Bowler [2010] found worse performance in the autistic group in measures of absolute difference, mean judgment ratio, and coefficient of variation. Finally, Sperduti, Pieron, Leboyer, and Zalla [2014] using a verbal estimation task reported comparable performance between autism and NT groups in terms of accuracy (Table 6).

**Table 6:** Prospective Estimation Task in ASC

		Sample		Task	Main conclusions	Commentaries
		ASC	NT			
Szelag et al. [2004]	*n*	7	7	Time reproduction	ASC group performed worse in the time reproduction task	Small sample Different IQ test in each group Trend to differences in IQ
	Age	12.6	Matched			
	IQ	82–102	95–145			
Gowen and Miall [2005]	*n*	12	12	Continuation and synchronization tapping	No differences in Coef. of variation ASC group showed greater absolute error and greater stimulus asynchrony on synchronization task	Small sample
	Age	24.2	24.2			
	IQ	114	114			
Wallace and Happe [2008]	*n*	25	25	Verbal estimation, production, and reproduction	No differences in time reproduction, time production, and time estimation	Recruitment of savants and a modification in the experimental paradigm could have been a factor affecting the results
	Age	14.1	13.84			
	IQ	96.36	100.08			
			
Martin et al. [2010]	*n*	20	20	Time reproduction	ASD group worse on measures of; absolute difference, mean judgment ratio, and mean coefficient of variation	No control of chronometric counting
	Age	35	35			
	IQ	106	108			
Maister and Plaisted‐Grant [2011]	*n*	21	21	Time reproduction	Differences in short (0.5 sec) and long durations (45 sec) Short‐term memory was correlated with the error scores in short durations between 1 and 10 sec	No data about over or underestimation Trend to differences in IQ
	Age	11.3	10.7			
	IQ	105.6	115.8			
Brenner et al. [2015]	*n*	27	25	Time reproduction	Poorer accuracy and consistency in ASC group Accuracy was found associated with age and consistency with working memory	
	Age	12.68	13.41			
	IQ	101.31	106.96			
Karaminis et al. [2016]	*n*	*n* = 23	*n* = 78	Time reproduction and discrimination	ASC group performed similar to younger children (6–7 years old) Less use of priors in ASC ASC children less accurate, but equally precise (consistent) in time reproduction task	Child friendly paradigm. The authors suggest using the traditional paradigms in order to avoid this possible interference
	age	Age: 12	(6–32 years old)			
	IQ	IQ: 100.03				
	
Sperduti et al. [2014]	*n*	15	17	Verbal estimation	Comparable reproduction error between groups	Small sample
	Age	33.53	33.06			
	IQ	109.38	105			

#### *Summary of prospective timing in autism*

As with temporal sensitivity, studies on prospective time involve a wide variety of methodologies and sample characteristics. Comparison methods have produced mixed results with indices showing differences between groups in some durations but not in others, and in all the studies there is at least one index showing no differences between groups. In children, two studies (out of three) show differences between groups in some indices, and in adults one study show differences and one report comparable performance. Altogether, the evidence from studies using comparison methods of prospective timing do not allow us to conclude a generalize impairment in these abilities in autism. Although, vulnerability in the abilities required by these tasks cannot be ruled out and in fact three out of five studies show differences between groups. More research is needed to identify which processes do or do not differ from the general population.

In estimation methods, the findings are also mixed although they tend to show differences between groups. In children, four out of five studies show worse performance in autism, while in adults two out of three studies show at least one measure of reduced performance in autism. In addition, in general the autistic group tends to show greater variability in their responses. It is important to note that many of the estimation studies make use of reproduction paradigms involving motor abilities that are absent in other paradigms, adding an additional variable that could be affecting the performance of the autistic sample in ways that are not measured or controlled.

Surprisingly, no studies have used comparison and estimation methods in the same sample, which would allow relationships to be established between the indices from the computer models in comparison methods, and the estimation methods indicators, which have the comparative leverage of being a perception in the same physical units the stimulus is defined. It is worth noting that the studies which have investigated effects of memory on timing performance (extracted from computer modeling or correlated with other tasks) suggest that memory impacts on prospective timing judgments in autism.

## Higher Level Temporal Processing

This section discusses the capacity to think about time as an abstract concept, where events take place within it, and the ability to be aware of one place in time and plan for events in the future. These set of processes are related to other complex cognitive processes such as episodic memory (e.g., when assigning temporal order to memories) and executive function (e.g., planning future actions, or managing information to do things “on time”), and the tasks involve perception of durations, meaning, and management of time in a range of minutes, hours, days, or even years. Possible impairment associated with these abilities may lead to difficulties in giving continuity to one's own experience. For instance, not knowing the temporal order of previous events would have consequences in assigning cause–effect relationships between your past experiences and your current behavior, and in your ability for planning future events using current and past information.

### *Time‐Based Prospective Memory*

Time‐based prospective memory (TBPM) is the ability to remember to execute a previously planned action at a previously defined moment [Williams, Boucher, Lind, & Christopher, 2013]. It has been hypothesized that autistic people have problems with this ability because of the high demands on executive function these tasks require. Five studies have researched TBPM in autism differentiating between time‐based and event‐based prospective memory (EBPM: remembering to behave in a specific manner when a previously defined cue is present in the environment).

Altgassen, Koban, and Kliegel [2012] compared 25 NT and 25 autistic adults' performance in the *Dresden Breakfast task* measuring TBPM and EBPM. Participants were asked to prepare breakfast for hypothetical visitors, so they needed to remember to take out the tea bag after 3 min in the cup or to put the butter in the table 6 min prior the arrival of the guests. If participants did these tasks with +‐60 sec, they were scored as correct. In addition, they recorded how many times they looked at the clock when performing the task. Autistic participants performed worse in both TBPM and EBPM tasks. This study also found a relationship between executive function and TBPM (but not with EBPM). Kretschmer, Altgassen, Rendell, and Bölte [2014] also found worse performance in the autistic group, but using a different task (virtual week prospective memory task). The third study in autistic adults [Williams, Jarrold, Grainger, & Lind, 2014] also reported diminished TBPM in autism, but comparable performance in EBPM. In the studies with children, Williams et al. [2013] and Henry et al. [2014] found impaired abilities of TBPM in autism but conserved EBPM, although different tasks were used in each study (Table 7).

**Table 7:** High‐Level Time Processing in ASC

		Sample		High‐level time processing ability	Tasks	Findings	Commentaries
		ASC	NT				
Altgassen et al. [2012]	*n*	25	25	Time‐based and event‐based prospective memory	Dresden breakfast task	Autistic group performed worse in both tasks	Clock was available to be checked
	Age	21.8 (6.68)	21.8 (6.06)				
	IQ	>85	–				
						Relationship between TBPM and executive function	
					
					
Kretschmer et al. [2014]	*n*	27	27	Time‐based and event‐based prospective memory	Virtual week prospective memory task	Autistic group performed worse in both tasks	Clock was available to be checked
	Age	35.63 (10.12)	39.85 (8.50)				
	IQ (Raven)	40.81	40.58				
					
Williams et al. [2014]	*n*	17	17	Time‐based and event‐based prospective memory	Word recognition task	Autistic group performed worse in TBPM, but comparable in the EBPM	Clock was available to be checked
	Age	31.06 (9.64)	31.92 (14.17)				
	IQ	114.06 (15.16)	117.71 (13.05)				
				
Williams et al. [2013]	*n*	21	21	Time‐based and event‐based prospective memory	2D computer‐based driving game	Autistic group performed worse in TBPM, but comparable in the EBPM	Clock was available to be checked
	Age	10.60 (2.01)	10.59 (1.31)				
	VIQ	103.57 (17.88)	106.48 (14.01)				
	PIQ	110.19 (16.35)	107.48 (13.23)				
Henry et al. [2014]	*n*	30	30	Time‐based and event‐based prospective memory	Virtual week prospective memory task	Autistic group performed worse in TBPM, but comparable in the EBPM	Clock was available to be checked
	Age	10.1 (1.47)	10.0 (1.46)				
	IQ	112.93 (16.71)	115.3 (14.69)				
Bennetto, Pennington, and Rogers [1996]	*n*	19	19	Memory for temporal order	An adaptation of the Corsi memory task	Autistic group perform worse for words and pictures	Comparison group was a mix of individuals with non‐autistic learning disabilities
	Age	15.95 (3.3)	15.23 (2.6)				
	IQ	88.89 (11.1)	91.74				
Gaigg, Bowler, and Gardiner [2013]	*n*	22	22	Memory for temporal order	Historic figures task	Autistic group showed difficulties in the order of episodic, but not semantic memory	Differences in executive function and attention
	Age	37.6 (13.4)	40.5 (10.8)				
	IQ	103.4 (13.4)	107 (16.4)				
Boucher, Pons, Lind, and Williams [2007]	*n*	23	23	Diachronic thinking	Tendency, transformation, synthesis	Autistic group was impaired in the three measures	
	Age	12.6 (2.3)	12.3 (2.25)				
	IQ (raven)	29 (5.3)	27 (5.4)				
	*n*	15	15	Diachronic thinking	Tendency, transformation, synthesis	Autistic group was impaired in the three measures	
	Age	14.3 (1.83)	14.6 (1.5)				
	IQ (raven)	26.4 (4.5)	23.7 (6.3)				

TBPM findings are highly consistent across studies with all the studies showing differences between groups. A limitation common to all of these studies is that these tasks assessed the ability to follow an instruction at designated times, but because the participants had the option of looking at a clock, it is very difficult to know if the differences between participant groups are due to a pure TBPM issue, or if they respond to problems with executive function (e.g., monitoring). Future research should measure prospective time tasks to assess the possible effects of basic timing abilities on TBPM.

### *Temporal Planning, Memory for Temporal Order, and Diachronic Thinking*

Three other studies have approached high‐level temporal processing issues in autism. In Allman et al. [2011] discussed earlier, the parents of the participants were given a “Parental time questionnaire,” modified slightly from the “It's About Time” questionnaire [Barkley, 1998]. The test contains such questions as: “How often does your child ask questions about their past?,” “How often does your child refer to a watch or clock in planning how much time he or she has left to do something?,” “How often does your child talk about or seem to think about what he/she will be doing tomorrow?.” Overall, the mean score for the autistic participants was significantly lower than the comparison group.

Bennetto et al. [1996], investigated autistic children and adolescents and compared them with a clinical comparison group with non‐autistic learning disorders using a task of *memory for temporal order*, which is the ability to give the correct temporal order to events already located in either long‐ or short‐term memory (differing from TOJs which are an immediate perceptual judgment). The autism group performed worse than controls for both pictures and words suggesting they were less able to represent temporal order in memory. In adults, Gaigg et al. [2013] studied the temporal order allocated to well‐known historical figures, finding difficulties in the order of episodic memory, but not in semantic memory (a class of memory that does not imply a temporal dimension). The authors acknowledge that the differences could be due to executive function and attentional issues, although that does not discard the presence of episodic memory difficulties.

Finally, Boucher et al. [2007] researched *diachronic thinking*, defined as “the propensity and capacity to think about events spreading across time.” The authors took the work from Montangero and colleagues who had investigated the development of diachronic thinking in NT children (Montangero, Pons & Scheidegger, 1996; Pons & Montangero, 1999). They had identified three components of this type of thinking: *Tendency* (“the tendency to think 'backwards' and 'forwards' across time”), *Transformation* (understanding that qualitative and quantitative changes can take place over time), and *Synthesis* (the ability to conceive of several distinct events forming parts of an overall whole). In two different studies, one in children and one in adolescents, Boucher et al. [2007] reported worse performance in autism compared to controls.

### *Summary of Higher Level Temporal Processing*

The evidence in higher order timing consistently shows impaired abilities in autism (all the studies point into the same direction), in comparison to low order timing as shown in time sensitivity and interval timing (mixed findings). However, replication is needed since the number of studies is small for some of these abilities, and due to the tasks used in these studies it is very difficult to disentangle the processes related to time perception from other cognitive abilities like memory and executive function. Future studies should attempt to address this issue and may use strategies such as those used in interval timing, computer modeling [Allman et al., 2011], or relating timing performance with memory abilities [Maister & Plaisted‐Grant, 2011]. It would be useful to measure these processes in conjunction with measurements on the more fundamental/lower order timing tasks to see how (or if) they map on to each other and/or on to other traits of autism.

## Conclusions

Autism involves a complex profile of cognitive differences across attention [Keehn, Lincoln, Müller, & Townsend, 2010; Keehn, Müller, & Townsend, 2013], social cognition [Dawson et al., 2004], and working memory [Kercood, Grskovic, Banda, & Begeske, 2014]. This review aimed to provide more clarity regarding whether the time perception difficulties often reported in are due to impairment in basic timing mechanisms, or are consequences of other cognitive impairments in autism. To this end, we systematically reviewed the scientific literature involving explicit measurements of time perception abilities in autistic population. The selected articles were categorized in three main clusters of time perception ability: temporal sensitivity, interval timing, and high‐level temporal processing. It remains unclear as to whether atypical timing is characteristic of autism, at least in terms of differences in the function of the internal clock. Findings from the literature revealed inconsistent findings, with a trend of finding differences in some tasks, but not in others. Divergent performance appears to be more commonly observed where tasks place demands on other, non‐timing cognitive processes and are less consistent in studies of fundamental or “pure” time perception abilities (tasks with less involvement of other cognitive resources). For example, in the studies of temporal thresholds, three out of five studies showed comparable performance between groups. In contrast, TBPM (i.e., a task that involve more complex cognitive demands), all the studies show evidence of impaired abilities in autism. So, while autistic people may or may not have problems distinguishing the durations of two stimuli such as two beeps (or some may have issues while other autistic individuals do not), the evidence shows that they may show issues with instructions such as “we will lunch in *five* minutes” (a TBPM task). A previous review by Allman and Falter [2015] proposed a similar explanation, but circumscribed to the supra‐second range as time judgment would get “worse as duration increases into the bounds of secondary executive function (working and episodic memory, sustained attention)”(p. 52).

We have argued that the differential consistency between the three levels could be explained by the differential cognitive load their tasks demand. Time sensitivity is mainly determined by a perceptual mechanism (depending on the sensory modality) and attention (except in MMN in EEG studies), with low participation of other processes such as working memory and no involvement of long‐term reference memory or executive function. Prospective interval timing tasks as explained by SET model have demands of attention, working and long‐term reference memory, and decision making. TBPM, where all studies showed differences between groups, adds a strong demand of executive function, since participants need to take decisions while multitasking, plan and adhere to a plan, inhibit behavior, and switch their attention between different stimuli. Therefore, it is possible that the differences in consistency between studies are anchored in those non‐timing cognitive processes, and not in an atypical “pure” time perception mechanism.

The studies in temporal sensitivity reveal informative trends about how autistic people distinguish between the temporal characteristics of stimuli in the environment. Temporal thresholds findings are mixed in children and adults, which could be explained by the different methodologies used in these five studies. This lack of consistency in the findings between studies should encourage replication studies, and make us question how robust the measurements that we are applying are, or how comparable the different methodologies to estimate thresholds are. Studies in TOJ, SJ, and temporal integration of multisensory information (although showing mixed results) tend to more consistently report atypicalities in autism. The studies reporting atypicalities in temporal integration of audiovisual stimuli in speech [Bebko et al., 2006; Irwin et al., 2011; Grossman et al., 2015; Stevenson et al., 2014; Noel et al., 2016] are consistent and might be related to the difficulties in language development, a common reported comorbidity in autism. Interestingly, language and communication symptomatology correlated with atypical performance in two studies of interval timing [Allman et al., 2011; Falter, Noreika, et al., 2012].

As shown by the EEG studies, atypicalities in autism show impaired abilities in childhood, but enhanced abilities in adulthood, suggesting a possible differential developmental trajectory for duration, since other auditory features as pitch have been described as enhanced in both children and adults [Kujala et al., 2007]. Taking into account that learning processes are likely to mediate those developmental trajectories, and that cross‐modal temporal processes improve with practice and training in the general population (Powers, Hillock & Wallace, 2009), future research could investigate how trainable these abilities are, and the possible impact of a program to train time on the social and non‐social atypicalities that characterize autism.

In the interval time studies, two studies concluded that autistic children performed similarly to younger NT children [Allman et al., 2011; Karaminis et al., 2016], which is consistent with the findings of temporal sensitivity suggesting a differential developmental trajectory in autism. One factor that may affect this differential developmental trajectory is working memory, a skill that has been shown to have strong age‐related components [Bayliss, Jarrold, Baddeley, Gunn, & Leigh, 2005] and that is in the core of SET model (thus affecting performance in interval timing tasks). In addition, working memory has shown an association with the performance of autistic individuals in interval timing tasks [as in Allman et al., 2011; Brenner et al., 2015]. Further research using computer modeling should involve a control task making judgments about another, non‐time related stimulus dimension (for instance pitch; see Harrington, Haaland, & Hermanowitz [1998]) in order to provide stronger evidence regarding possible atypicalities in each SET model component. Indeed, atypicalities in integration rather than impairment in basic processes (as a pure perceptual issue could be) have been proposed in autism in other areas (for an example in sensory‐motor integration, see Gowen and Hamilton, 2013).

An area of interval timing where our systematic search showed no results was retrospective timing, although after the time limits of this systematic review there is one study including retrospective timing data [see Isaksson et al., 2018]). Retrospective timing is the judgment made when the participant is asked an unexpected question about a duration. For example, if you were asked how long have you been reading this document, you did not know at the start of reading that you would be asked this, so you could not have started your clock mechanism. People are able to make such duration judgments with some accuracy, although considerably less than for prospective timing [Hicks, 1992]. To date there is a paucity of retrospective timing studies even in NT populations, mainly due methodological problems as once participants have completed one trial then they are alerted that timing judgments are required, and any further judgments will be prospective. However, this would be a fruitful area of research in ASC.

Different aspects of high‐order temporal processing have been researched, with consistent findings of atypicalities in autism. Tasks like “Dresden breakfast task” used in TBPM have an ecological validity, and future approaches could complement such measurements with fundamental timing tasks in order to relate them to high‐order time processing. Additionally, there are related processes that have not been researched at all, such as passage of time judgments (how quickly time seems to pass) and temporal processing and information processing rates. The latter is interesting, since work in NT population is suggesting that there is at least a strong correlational (perhaps causal) relationship between the rate of the internal clock and the rate at which people can process information, with faster information processing rates associated with higher internal clock speeds [Droit‐Volet & Zélanti, 2013; Jones, Allely, & Wearden, 2011].

When reviewing the literature on time perception in autism there are two related issues across the categories of timing tasks, which are likely to contribute to the variability in findings. First, studies tend to use small sample sizes. This is part of a wider issue with power and replicability in the psychological sciences [see Button et al., 2013], but is likely to be particularly problematic when attempting to make inferences about a heterogeneous condition such as autism. Second, the literature utilizes a variety of different methodologies (in terms of procedures and data analysis). As consequence, it is difficult to directly compare results in autism studies with previous research in NTs. For instance, a frequent issue in studies working with a supra‐second range is chronometric counting, which normally violates scalar timing as subdividing the duration into smaller units makes the timing of longer durations less variable than for shorter ones (the opposite to the Scalar property). Although some studies [as Martin et al., 2010] have addressed this, not all studies have done it, or they use different methods to do so, making the direct comparison between studies difficult. These methodological differences are likely to contribute to the mixed findings previously discussed in time sensitivity and interval timing.

There is a remaining question about whether deficits in any type of timing actually have any importance in terms of autistic symptoms (for a discussion about possible links see Boucher [2001], Allman & DeLeon [2009], and Allman [2011]). A related question is how enhanced time perception abilities impact everyday life activities in comparison with impaired abilities. A possible question that can be addressed in future research is whether autistic people follow a different developmental trajectory in their time perception abilities. In addition, it is unknown (even in the NT population) how abilities or deficits in different types of timing map on to each other (if at all). So, do problems in fundamental timing processes predict problems with higher order processing of time and/or vice versa? This would be a fruitful avenue of investigation as the results would be of value in both understanding how deficits in timing predict/cause atypicalities in other cognitive processes and in everyday activities in autism, and how performance in different types of timing map on to each other in the general population, which remains largely unexplored. Finally, a limitation of this systematic review was the omission of the concept “timing” in the systematic search. The reason of its exclusion was that “timing” is a very wide concept that is used to refer to many different processes other than time perception. Nevertheless, we included concepts and methodologies (see methods section) that are used in time perception research in the search, decreasing the likelihood of missing relevant studies.

In summary, previous research has attempted to characterize time perception in autism, but important questions remain unanswered. Our classification of the timing tasks in three hierarchical levels has revealed a different pattern of results at each level. This raises a question about this differential vulnerability autistic individuals have for each level of complexity. A possible explanation is that the fundamental timing mechanism in terms of an internal clock is preserved in autism: if one of the main differences between the three levels is their complexity in the cognitive resources needed, then the differences could be explained by the involvement of those other cognitive processes. The strategy we propose for resolving these issues follows two main principles: (a) to assess at least one measurement of each level of time perception in the same sample avoiding modifications of the original task (e.g., time sensitivity thresholds; interval timing by estimation and comparison methods—verbal estimation and temporal generalization; retrospective timing; TBPM; memory for temporal order); (b) to make use of computer modeling in order to explore any specific atypicalities in the pacemaker, memory, or decision making stage of SET model (involving at least one non‐timing control task, e.g., pitch).

Characterizing time perception abilities in autism by working with a taxonomy of timing abilities (time sensitivity, interval timing, and high‐level time processing) would improve precision in how timing is measured and should encourage attempts to replicate findings at each level, avoiding the generalization of findings from one level to another level. Finally, having a characterization of each level as a separate process will facilitate the future design of targeted interventions, if they are needed.

## Conflict of Interest

The authors have no conflicts of interest to declare.
